# Comprehensive inventory of protein complexes in the Protein Data Bank from consistent classification of interfaces

**DOI:** 10.1186/1471-2105-9-234

**Published:** 2008-05-12

**Authors:** Andrew J Bordner, Andrey A Gorin

**Affiliations:** 1 Computational Science and Mathematics Division and BioEnergy Science Center, Oak Ridge National Laboratory, P.O. Box 2008, MS 6173, Oak Ridge, TN 37831, USA; 2 Mayo Clinic, 13400 East Shea Boulevard, Scottsdale, AZ 85259, USA

## Abstract

**Background:**

Protein-protein interactions are ubiquitous and essential for all cellular processes. High-resolution X-ray crystallographic structures of protein complexes can reveal the details of their function and provide a basis for many computational and experimental approaches. Differentiation between biological and non-biological contacts and reconstruction of the intact complex is a challenging computational problem. A successful solution can provide additional insights into the fundamental principles of biological recognition and reduce errors in many algorithms and databases utilizing interaction information extracted from the Protein Data Bank (PDB).

**Results:**

We have developed a method for identifying protein complexes in the PDB X-ray structures by a four step procedure: (1) comprehensively collecting all protein-protein interfaces; (2) clustering similar protein-protein interfaces together; (3) estimating the probability that each cluster is relevant based on a diverse set of properties; and (4) combining these scores for each PDB entry in order to predict the complex structure. The resulting clusters of biologically relevant interfaces provide a reliable catalog of evolutionary conserved protein-protein interactions. These interfaces, as well as the predicted protein complexes, are available from the Protein Interface Server (PInS) website (see Availability and requirements section).

**Conclusion:**

Our method demonstrates an almost two-fold reduction of the annotation error rate as evaluated on a large benchmark set of complexes validated from the literature. We also estimate relative contributions of each interface property to the accurate discrimination of biologically relevant interfaces and discuss possible directions for further improving the prediction method.

## Background

Proteins usually accomplish their biological functions as components of stable complexes or through transient interactions with other proteins. The most detailed experimental information on protein complexes comes from high-resolution X-ray structures. These structures provide clues on the mechanism by which the complex accomplishes its function, give insights into the physical and evolutionary principles of protein-protein interactions through statistical analysis [1-4], and can be used as templates for the computational prediction of protein-protein interactions using docking [5] or threading [6,7] techniques.

Because protein crystals contain a regular array of molecules it is a nontrivial task to determine which molecules form the biologically relevant complex. Only a subset of the molecules in the asymmetric unit or additional molecules related by space group transformations may be included in the complex. Although Protein Data Bank (PDB) files contain information on generating the biological complex (BIOMT records), this information is error prone. An approximate lower bound, which is discussed in the Results section, suggests that the error rate for BIOMT annotation is at least 9%. This means that structural studies, such as those mentioned above, must depend on either small but reliable manually curated sets of protein complex structures or on large but significantly less reliable automatically generated sets.

Computational methods that predict the biological complex for an X-ray structure can improve the reliability of complex annotations. The earliest methods were applied only to the more limited problem of distinguishing homodimers from monomers by using a variety of differentiating features: interface area and atomic pair contacts [8], fraction of surface residues in the interface and evolutionary conservation [9], and a combination of non-polar interface area, fraction of buried interface atoms, and residue propensity score [10]. A more general method, described in [11], used scores based on atom pairs that contact across interfaces combined with iterative partitioning of the graph representing crystal contacts in order to predict complexes. This method gave a 16% error rate on a non-redundant set of 218 X-ray structures. The Protein Quaternary Structure (PQS) server [12,13] discriminates biological and crystal contacts based on a weighted score computed from the interface accessible area, number of interface residues, solvation energy, and the number of salt bridges and disulphide bonds. Finally, a new prediction method and web server, PISA [14,15], was introduced during the course of this work. That method used an empirical estimate of both enthalpic and entropic contributions to the binding free energy in order to predict stable complexes. Small ligands were also included in the free energy calculation. Although there are many online databases that contain analyses of protein complex structures obtained from other sources [16-22], such as BIOMT annotation or PQS, there are currently only two other databases that contain predictions of complexes for the entire PDB, namely PQS and PISA. The lack of many independent prediction databases is probably due to the difficulty in predicting complete protein complexes and is one motivation for this work.

We have developed a new method for identifying biological relevant protein complexes in X-ray structures and demonstrate a significant reduction in the annotation error rate. In addition, our database (the Protein Interface Server (PInS), see Availability and requirements section for URL), contains a useful byproduct – a new classification of all protein contacts (biologically relevant and crystallographic) found in the PDB. First, protein-protein interfaces are grouped by similarity and all interfaces in a particular cluster are either all predicted to be specific (biological) contacts or non-specific (crystal) contacts. Crystal contacts are defined to be protein-protein interfaces that only occur in the non-physiological environment of the crystal (e.g. high protein concentration, low temperature, and compounds added to aid crystallization). Because all interfaces in a cluster containing biological interfaces are between proteins with similar amino acid sequences and have similar binding geometry, they presumably arose through evolutionary divergence. Thus they provide a valuable resource for studying how similar interfaces appear in different complexes of diverse functions. Second, the protein-protein interface clusters are classified by a machine learning method that utilizes a robust set of diverse interface properties. Previously we demonstrated that a similar set of properties was sufficient to distinguish near-native docked conformations in a large set of decoys [5]. The combination of a large set of properties and a more sophisticated statistical model is expected to yield more accurate interface predictions. Third, the prediction method incorporates all PDB structures (as we are using interface clusters across the PDB) rather that making a separate prediction on individual structures. This means that information from multiple structures is combined to make the prediction of each complex. Finally, we derive the overall complex structure by a rigorous probabilistic framework that combines the probability scores from the interface predictions with probabilities derived from the PDB annotation.

## Methods

### Benchmark set of protein complex structures

A non-redundant benchmark set of 435 complex structures was created by combining the sets of [11] and [3], removing redundant complexes, and adding an additional 200 unrelated complexes, where all annotations were manually verified from an extensive search of the literature. PDB entry 1MDA from the Ponstingl et al. 2003 set was not included because four chains (H, J, L, M) were found to have incorrect amino acid sequences. The 200 additional complexes were iteratively added by randomly choosing a complex such that no two complexes in the combined set had corresponding subunits with > 25% sequence identity. The best quality structure, with the fewest chain breaks and mutations and highest resolution, was chosen among all PDB structures of the same complex. Furthermore, the literature, starting from the original article for the X-ray structure, was examined in order to identify the structure of the relevant protein complex and to insure that the prevalent oligomeric state of the complex in solution was experimentally verified and agreed with the structure. This set was used for training and validating the Random Forest classifier for protein-protein interface prediction and for assessing the overall prediction accuracy for complexes. The set is available, together with the annotations and prediction results [see Additional file 1].

### Protein-protein interface prediction data

Protein-protein interfaces were predicted to be either biologically relevant contacts or crystal contacts using a Random Forest classifier [23] trained on diverse interface properties. The interface properties are the following: 210 contacting residue pair counts, 20 residue propensity log(p) values, evolutionary conservation log(p), interface area, number of intermolecular hydrogen bonds and disulfide bonds, packing density, homo- or hetero-interface, and symmetric or non-symmetric interface. Both the residue-level [4] and atomic-level properties [11] were previously shown to distinguish protein-protein interfaces. Contacting residues had at least one pair of non-hydrogen atoms, one in each residue, separated by less than 4 Å. Only interfaces with at least 5 intermolecular residue contacts were included in the prediction data. The residue propensity log(p) values reflect the number of each residue type occurring in the interface compared with the number expected from a random reshuffling of residue types on the surface. This results in a probability that is calculated from a hypergeometric null distribution, as described in [4]. The interface area was defined as 0.5(SASA of protein 1 + SASA of protein 2 – SASA of both proteins), in which the solvent accessible surface area (SASA) was calculated with the DSSP program [24]. The number of intermolecular hydrogen bonds was determined by adding hydrogen atoms and optimizing their geometry using the ICM program [25] and counting the number of potential hydrogen donor/acceptor pairs within 2.5 Å of each other. Intermolecular disulfide bonds were defined by two cysteine residues with S-S separations between 1.5 Å and 2.5 Å. The packing density was calculated as SASA_0_/(solvent excluded surface area), in which SASA_0 _is the SASA with a zero probe sphere radius and the solvent excluded surface is calculated using a 1.4 Å radius probe sphere. The evolutionary conservation of each residue was defined as the column entropy S for a multiple alignment of similar sequences

(1) $$S=-\sum_{i=1}^{20}f_{i}\log f_{i}$$

in which *f*_*i *_are the residue frequencies in the corresponding alignment column. The multiple sequence alignment was generated by collecting similar sequences from the NCBI nr database using BLAST [26] with the protein sequence of interest as a query and an E-value cutoff of 0.01, removing redundant sequences with > 90% sequence identity using the Cd-hit program [27], and finally aligning them using MUSCLE [28]. The evolutionary conservation p-value, which reflects the probability of observing more highly conserved residues in the interface than in the remaining protein surface by chance, was calculated with the Wilcoxon rank-sum test [29]. Finally, whether or not the interface was symmetric, *i.e*. possesses two-fold crystallographic symmetry, was included in the interface properties because it is a common feature of homodimers.

### Clustering similar interfaces

Similar protein-protein interfaces were clustered into groups. Essentially those groups are groups of identical (or almost identical) structures, where corresponding residues are globally aligned. All interfaces in a particular group are assigned to be either all crystal contacts or all biological interfaces (details below in the *Clustering procedure *section). Previous studies have used various criteria to cluster protein-protein interfaces by similarity [16,19,30], but one important innovation in our method is the use of such clusters to make consistent predictions of biological interfaces and consequently consistent predictions of biological complexes across the PDB.

### Assigning Pfam residue-residue contacts

PDB residue numbers are inconsistent in general, *i.e*. the same residue in different structures of the same protein may have different numbers. Because of this, they cannot be used directly in order to compare residue-residue contacts in different structures. Therefore residue numbers were made consistent by mapping them to either the corresponding Pfam [31] alignment column number or, if no Pfam assignment is possible, by the residue index of the most similar Uniprot sequence [32]. Hereafter the combination of the Pfam family accession number and alignment column number (or Uniprot accession number and sequence index) is denoted as the uniform residue number. This also insures that aligned residues in homologous protein sequences are designated by the same number. PDB sequences were first aligned to the most similar Uniprot sequence, which was determined by performing a BLAST search of Uniprot amino acid sequences and choosing the Uniprot sequence with highest sequence identity to the query PDB sequence. Next the correspondence between the Uniprot and Pfam sequence was deduced from the full multiple alignment of Uniprot sequences used to define each Pfam family. Because breaks in the protein sequence in a PDB structure may lead to alignment errors near the breaks, each contiguous segment of the protein chain was separately aligned to the corresponding Uniprot sequence using the EMBOSS program needle for global pairwise sequence alignment [33].

Next the set of contacting residue pairs (< 4 Å atom separation) in each protein-protein interface was calculated with the residues referred to by their uniform residue number. All original protein chains as well as symmetry-related chains within 25 Å of any of the original chains, with the inter-chain separation defined by the minimum distance between non-hydrogen atoms in each chain, were included in order to insure all interfaces for the complete biological complex are present. The symmetry-related molecules were generated using PyMOL [34].

### Clustering procedure

Interfaces were then clustered so that the minimum fractional residue contact overlap O, defined by

(2) $$O=\frac{N(\text{interface 1 contacts }\cap \text{ interface 2 contacts)}}{\min\left(N\left(\text{interface 1 contacts}\right)\text{,}N\left(\text{interface 2 contacts}\right)\right)}$$

was at least 0.3 between any two interfaces in the same cluster. *N*(interface *i *contacts) denotes the number of contacting residue pairs in interface *i *and the numerator is the number of common contacting residue pairs. This overlap cutoff value is actually a rather strict requirement as residues in the interfaces make multiple contacts with each other, so that when 30% of all contacts are the same between two interfaces it is very unlikely that the interfaces are unrelated. Although clustering a large number of interfaces would be computationally expensive, the clustering problem can be subdivided into manageable subproblems. First identical interfaces, with the same proteins and same relative space transformation, within each PDB complex were rapidly identified and grouped. Next, all non-identical interfaces throughout the PDB were clustered using hierarchical complete linkage clustering with distance measure 1-O and the largest clusters with O ≥ 0.3 were selected. This task was subdivided by clustering only groups of interfaces with the same pair of Pfam accession numbers since the overlap between interfaces with different Pfam numbers is zero by definition. This clustering reduced the 254879 non-identical protein-protein interfaces into 58274 clusters. Identical protein-protein interfaces, which have the same residue-residue contacts between the same proteins, have fractional residue contact overlap O = 1.0 and are therefore trivially assigned to the same cluster.

### Random Forest classifier

The Random Forest method uses the consensus prediction from an ensemble of randomized decision trees for classification [23]. It was chosen for interface prediction because of several desirable properties: resistance to overfitting, speed, the ability to use combinations of continuous and discrete data, and insensitivity to data normalization. The randomForest package in R was used [35,36]. Approximately 15% of the benchmark set interfaces had missing evolutionary conservation data because a sufficient number of homologous sequences (at least 20) could not be found. Missing data values were first imputed by the median value of that variable. The input data included all interface properties described in *Protein-protein interface prediction data*.

Each protein-protein interface was classified as either a specific contact appearing in a biological complex or a non-specific crystal contact. The variation of the Random Forest score within each cluster was estimated by calculating the standard deviation in the score within a sample of interfaces of intermediate size. All 93 clusters containing exactly 20 interfaces were chosen for this purpose. The average of the standard deviation in the scores within each cluster was found to be only 0.04. Because the intra-cluster score variation is so small and also to speed up the calculation, predictions were made for only a single randomly chosen interface in each cluster and all interfaces in the cluster were assigned to the same class, as described above. A total of 1000 classification trees generated from 20 random chosen variables were used in the Random Forest classifier. As expected [23], the prediction accuracy was largely insensitive to these model parameters (data not shown).

A Random Forest score was calculated as the fraction of trees that classify the interface as a biological interface. All other interfaces in the same cluster, which were not used with the Random Forest classifier, were then also assigned this score.

### Cross-validation procedure

Prediction performance was assessed using 10-fold cross-validation. The 2006 interface clusters appearing in the benchmark set were randomly divided into 10 approximately equal size test sets. Predictions were then made for each test set in turn using a Random Forest classifier trained on the remaining interface data in the other 9 sets and the prediction statistics calculated. This procedure prevents overly optimistic performance estimates due to overfitting. Predictions for structures not included in the benchmark set were made using a Random Forest classifier trained on data for all interface clusters in the benchmark set.

### Estimating the contribution of each interface property to prediction accuracy

One further advantage of the Random Forest classifier is the ability to quickly estimate the contribution of each input variable to the overall prediction accuracy. This is accomplished by calculating the average decrease in accuracy for data upon permuting the values for the variable of interest. This can be efficiently calculated because only the so-called out-of-bag data is used (data not included in the bootstrap sample) [23]. Because the benchmark set data is significantly unbalanced, the variable importance was calculated using a balanced set of data, containing all 436 specific interface examples and an equal number of randomly selected non-specific interface examples. The importance for two groups of variables, all 20 residue propensity log(p) values and all 210 contacting residue pair counts was calculated using an alternative procedure because importance for groups of variables is not implemented in the randomForest package. In this case, the 10-fold cross-validation accuracy was compared with the accuracy obtained by permuting the variables of interest in the cross-validation test data sets. The accuracy difference was then averaged over 100 independent calculations.

### Prediction of the protein complex

Overall, the prediction procedure for the protein complexes combines local information from the Random Forest interface prediction scores with global information from the number of subunits in the BIOMT complex using a consistent probabilistic framework. The prediction is performed by maximizing the total probability over all structures simultaneously so that, in general, information from multiple structures contributes to the prediction of each individual complex.

### Interface contribution to the total probability

**Figure 1:**
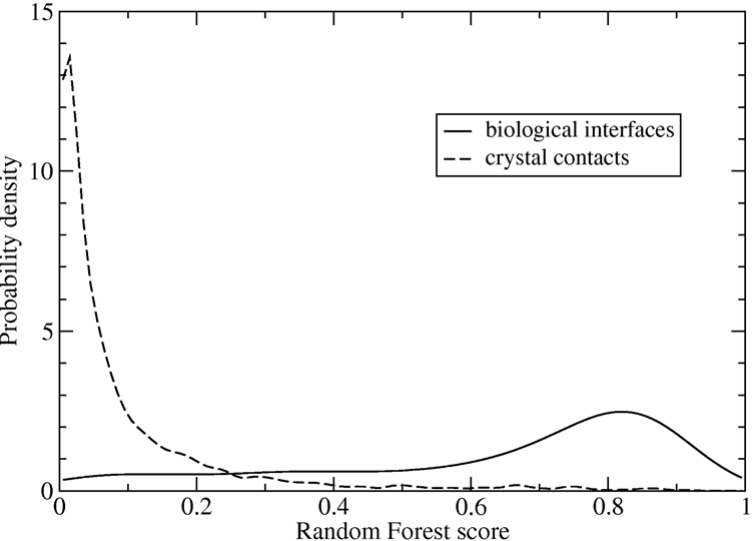
Estimated probability density function for the Random Forest scores in each class.

The Random Forest scores for each interface in a predicted complex need to be converted into probabilities in order to calculate the total likelihood that the complex is correct. This was accomplished by separately fitting the score distributions for biological interfaces (*P*_specific _(*S*)) and crystal contact interfaces (*P*_non-specific _(*S*)) in the benchmark set using gaussian kernel density estimation. The resulting smooth distributions are shown in Figure 1.

The local interface component of the score for each particular complex can be obtained for each possible interface assignment (specific or non-specific) by computing the following product over all unique interface clusters in the predicted complex.

(3) $$P\left(\left\{{\text{S}}_{\text{i}}\right\}\right)=\prod_{C_{i}\in \text{ specific}}P_{\text{ specific}}\left(S_{i}\right)\prod_{C_{i}\in non-\text{ specific}}P_{\text{ non-specific}}\left(S_{i}\right)$$

This equation assumes that the probabilities for each unique interface are independent. This is a very good approximation, because we cluster all similar interfaces together, and interfaces in a cluster are either all assigned as specific or all assigned as non-specific. It is not possible to consider as independent closely related interfaces within clusters.

### BIOMT contribution to the total probability

Even though the BIOMT annotation in the PDB files, which specifies how to generate the biological complex, is error-prone, it is still often correct and thus provides valuable information for predicting the complex. Therefore a predicted complex whose oligomeric state agrees with the BIOMT annotation should be assigned a higher likelihood to be correct than one with a different oligomeric state. We have chosen the number of subunits in the complex as a criterion of such agreement because often the only independent experimental information on the complex, aside from the crystal structure itself, is its oligomeric state as measured by, for example, chromatography.

The probability that the number of subunits in the BIOMT complex (*N*_BIOMT_) agrees with the number of subunits in the actual complex (*N*) is

(4) *P*(*N *= *N*_BIOMT_) = *P*(BIOMT correct) + *P*(*N*_BIOMT _| BIOMT correct)P(BIOMT incorrect)

If the probability of observing *N*_BIOMT _subunits by chance is estimated by the fraction of all possible complexes, resulting from all $2^{N_{\text{clusters}}}$ different possible interface assignments, that have *N*_BIOMT _subunits then Equation 4 becomes

(5) $$\begin{array}{l} P\left(N=N_{BIOMT}\right)\approx P\left(\text{BIOMT correct}\right)\\ +\left(1-P\left(\text{BIOMT correct}\right)\right)\frac{N\left(N_{BIOMT}\;\text{subunits}\right)}{\sum_{\text{i}}N\left(i\;\text{subunits}\right)} \end{array}$$

in which *N*(*i *subunits) is the number of predicted complexes with *i *subunits out of all possible predicted complexes.

There are two different ways in which *N *can agree with *N*_BIOMT_: either the BIOMT complex is correct (first term in Equations 4 and 5) or the BIOMT complex is incorrect and the number of subunits agrees by chance (second terms in Equations 4 and 5). The value of *P*(BIOMT correct) is approximated by its upper bound due to consistency (0.91) discussed above. The BIOMT contribution, *P*_BIOMT_, to the overall likelihood of the predicted complex can be then calculated based on the number of unique subunits observed in the prediction as *P*(*N *= *N*_BIOMT_) from Equation 5, if *N *= *N*_BIOMT_, or 1 - *P*(*N *= *N*_BIOMT_), if *N *≠ *N*_BIOMT_.

### Total probability for each predicted complex

The probability for a particular complex is calculated as the product of the BIOMT contribution, *P*_BIOMT_, (reflecting global information captured in Equation 5) and the local interface contribution, *P*({*S*_*i*_}), in Equation 3. The total likelihood of a particular interface assignment is the product of the probabilities over all complexes considered.

The set of protein complex subunits and resulting protein complexes may be represented by a graph in which each node corresponds to a particular subunit and edges join contacting subunits. There are two types of edges in the graph: specific (*i.e*. biological contact) or non-specific (*i.e*. crystal contact) interfaces between the two corresponding subunits. For a particular assignment of the interfaces, the component of the graph that (1) is connected by specific contacts, (2) contains at least one subunit in the asymmetric unit, and (3) contains all unique subunits is the predicted complex. Because the interfaces in a cluster are considered either all specific or all non-specific there are $2^{N_{\text{clusters}}}$ different possible interface assignments in which *N*_clusters _is the total number of clusters.

A constraint is imposed so that each predicted complex is required to include each non-redundant protein chain in the X-ray structure at least once. This is required because presumably each structure contains a single biologically relevant complex and not a collection of proteins that form multiple non-interacting complexes. In the few cases in which a consistent assignment of interfaces clusters that satisfies this constraint does not exist, only the assignments that minimally violate the constraint (have the fewest structures that violate it) are considered.

### Prediction of protein complexes using maximization of the total likelihood

The assignment of interface clusters as either specific or non-specific and the resulting predictions of biological complexes are then accomplished by maximizing the total likelihood over all possible interface cluster assignments subject to the aforementioned constraint. Because there are 58274 clusters and the number of possible interface assignments is $2^{N_{\text{clusters}}}$ (for each interface specific or non-specific), it is fortunate that the optimization problem can be broken down into independent subproblems.

This is done by generating a graph in which each node represents a cluster and edges are placed between two nodes for clusters co-occurring in any structure. Next, all connected components in this graph are determined. Independent optimizations are then performed for each set of interface clusters corresponding to nodes present in a single connected component. The optimization was done by exhaustive enumeration of all interface cluster assignments in subproblems with ≤ 15 interface clusters and otherwise using a Monte Carlo algorithm employing Metropolis sampling with a temperature parameter of 0.4. The vast majority of subproblems (92%) could be exactly solved by the former method.

## Results and Discussion

### Protein-protein interface prediction performance

**Figure 2:**
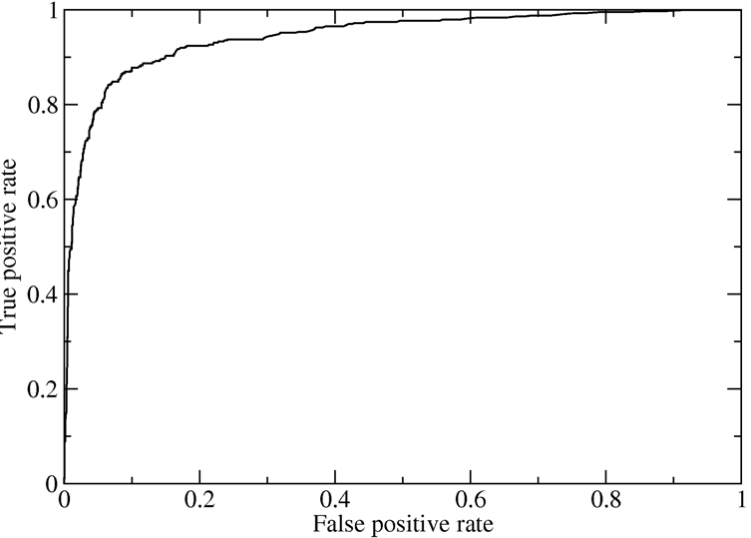
**ROC curve for the Random Forest prediction of benchmark set interfaces using 10-fold cross-validation**. The high prediction accuracy is shown by an area under the curve as high as 0.945.

The performance of the Random Forest classifier in predicting protein-protein interfaces was assessed by plotting the Receiver Operator Characteristic (ROC) curve for the 10-fold cross validation results on the benchmark set. The ROC curve, shown in Figure 2, displays the tradeoff between prediction sensitivity and specificity. The Random Forest score cutoff is the parameter that is varied along the curve. The value of 0.945 for the area under the ROC curve is near the maximum value of 1.0 and demonstrates the high prediction accuracy.

### Contributions of interface properties to prediction accuracy

As described in the Methods section, the importance of each interface property to the overall Random Forest prediction accuracy can easily be calculated. This can give insight into which properties are most useful in discriminating specific protein-protein interfaces from non-specific interfaces and provide guidance for future prediction efforts.

First we compare the relative importance of individual properties using the out-of-bag data. The properties making the largest contribution to the prediction accuracy, along with their relative importance, are listed in Table 1.

**Table 1:** Relative importance of interface properties to the Random Forest accuracy for discriminating specific and non-specific protein-protein interfaces.

Interface Property	Relative Importance
interface area	1.0
number of intermolecular hydrogen bonds	0.553
210 contacting residues pair counts*	0.224
20 residue propensities*	0.184
evolutionary conservation	0.168
leucine residue propensity	0.104
phenylalanine residue propensity	0.0950
symmetric/non-symmetric interface	0.0896
isoleucine residue propensity	0.0753
packing density	0.0665
tyrosine residue propensity	0.0619
number of leucine-valine contacts	0.0570
Number of leucine-glutamine contacts	0.0500

All properties with a relative importance of at least 0.05 are shown. An asterisk indicates a group rather than individual properties.

The interface area makes the largest contribution to the prediction accuracy. Its importance in discriminating specific protein-protein interfaces has been noted in earlier studies [8,12,37]. Of course, any criteria used to select which interfaces are included in the training data will affect the relative contribution of each interface property to the prediction accuracy. As mentioned in the Methods section, all interfaces were required to have at least 5 intermolecular residue contacts. This excludes many interfaces with low interface area, but is still quite permissive. If the cutoff on the number of residue contacts were increased or if a high cutoff on the interface area were applied, it is expected that the importance of the interface area would decrease because this would exclude small interfaces with low surface area, most of which are non-specific. Although specific interfaces in dimers typically have an area of at least 350 Å^2 ^[38], smaller interfaces are often present in higher order complexes. The motivation for applying a relatively lax cutoff on interface size is to avoid removing small specific interfaces and to allow the Random Forest classifier to select the specific interfaces based on interface area, in addition to the other interface properties, rather than employing a hard cutoff.

The number of intermolecular hydrogen bonds gives the second largest contribution (55%) to the prediction accuracy. This is expected since it is highly correlated with the interface surface area [38,39], with a correlation coefficient of 0.89 for the benchmark set data. Hydrogen bonds provide both favorable energetic contributions as well as specificity to the interaction [40].

The 210 residue contacting pair counts and the 20 residue propensities, each considered as groups of variables, contributed 22.4% and 18.4%, respectively. Examining the importance the propensities for each residue type reveals that the largest contributions are from leucine, phenylalanine, isoleucine, tyrosine, and valine, in decreasing order of importance. A previous study that used the same statistic as a measure of the propensity of particular residue types to appear in protein-protein interfaces also found that these were some of the most prevalent in interfaces [4]. The contacting residue pairs that contributed the most to the prediction accuracy were L-V, L-Q, L-L, D-R, A-Y, L-Y, V-V, F-I, R-Y, and L-P, in decreasing order of importance. All of these contacts involve at least one of the important residue types, except for D-R, which can potentially form a salt bridge.

### Protein complex prediction performance

The performances of different prediction methods were evaluated by counting the number of correctly predicted protein complexes having the correct stoichiometry, or oligomeric state. A total of 46 out of the 435 benchmark set complexes were incorrectly predicted, resulting in an error rate of only about 11% for our method. The error rate for the subset of 214 benchmark set complexes taken from the Ponstingl et al. 2003 set was 13%. This is slightly lower than the 16% error rate reported in that study. We also calculated the error rates for PQS and PISA predictions for comparison. One potential difficulty with this is that both of these databases contain multiple predictions for some PDB entries. This is not a significant problem for PQS since only 3 of the 75 PDB entries in the benchmark set with multiple predicted complexes had different oligomeric states, which is the basis for our evaluation of prediction results. Only the most stable predicted complex for each PDB entry was used to calculate the error rate for PISA, and furthermore only the total number of subunits in each complex was compared, because only these results were available for automatic download. There were between 75 and 78 incorrect predictions for PQS, depending on which of the multiple predicted complexes were chosen for comparison, yielding an error rate of approximately 17%–18%. A total of 104 of the predicted PISA complexes disagreed, giving an error rate of approximately 24%. Unfortunately, this value is not directly comparable with that for our method for the reasons mentioned above. However, the PQS error rate is significantly higher, even after accounting for the few PDB entries with multiple predicted structures.

**Figure 3:**
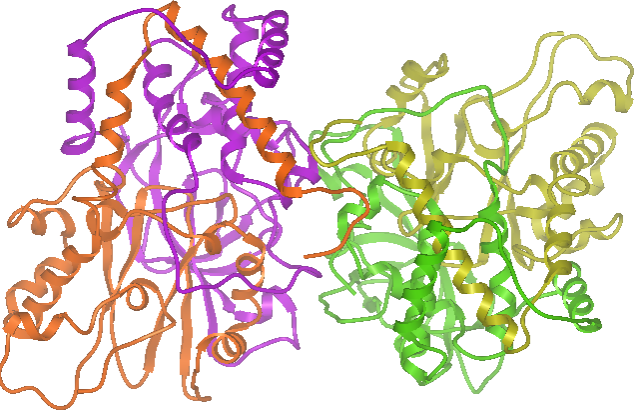
Correct predicted α_2_β_2 _structure for a bacterial nitrile hydratase from a PDB structure (entry 1UGQ) that is incorrectly annotated as a heterodimer.

A comparison of the predicted protein complexes with those generated from the PDB annotation revealed that a total of 17% of the predicted complexes are different, *i.e*. have different stoichiometry. A further breakdown of these complexes by type is shown in Table 2. It is apparent that there are considerably more complexes that are predicted to be homomultimers but in the BIOMT record they are annotated as monomers than the converse. One possible explanation is that X-ray structures of a single protein are annotated as a monomer by default if no experimental information on their oligomeric state is available. Figure 3 shows an example of one such case in which the complex structure was successfully predicted but the PDB annotation was incorrect. Unfortunately, the large number of PDB entries with disagreements between the predicted complexes and PDB annotation precludes manual verification of their correctness.

**Table 2:** Number of PDB entries for which the stoichiometry of the predicted complex differs from that of the complex specified by the PDB BIOMT annotation.

Predicted complex	Complex from PDB BIOMT annotation	Number of PDB entries
monomer	homomultimer	678
homomultimer	monomer	2042
homomultimer	homomultimer	1029
heteromultimer	heteromultimer	1115

Total		4864

Each complex is classified as either monomer, homomultimer (> 1 identical subunits), or heteromultimer (> 1 non-identical subunits).

### Analysis of domain contacts in predicted biological complexes

**Figure 4:**
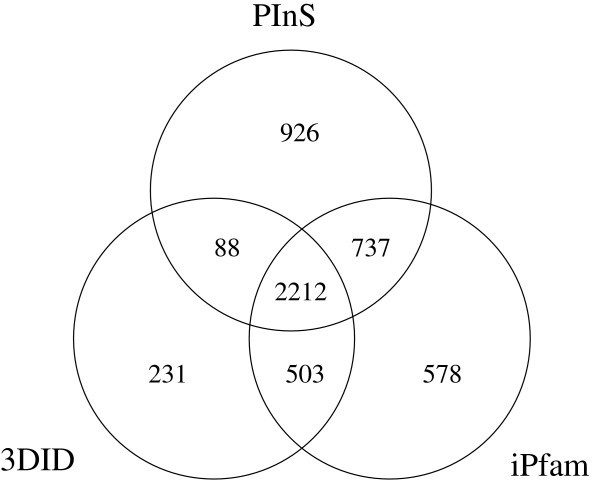
Venn diagram of the number of common Pfam-A domain family contacts in the predicted biological complexes, in the 3DID database, and in the iPfam database (version 21.0).

The prediction method for protein complexes also yields clusters of related protein-protein interfaces found in biological complexes. These predicted Pfam-A domain-domain contacts were compared with those in the iPfam [41] and 3DID databases. The overlap between the different sets of domain-domain contacts in each database is shown in Figure 4. This figure does not include the additional 4248 distinct Pfam contacts containing a Pfam-B domain that are present in the predicted biological complexes but not included in either iPfam or 3DID. Interestingly, Pfam domain-domain contacts are predicted very differently in the iPfam and 3DID databases. This difference alone is a strong indication that further analysis is needed.

Indeed, the iPfam database includes all contacts, both specific and non-specific and the 3DID database used an empirical residue contact score to remove possible non-specific contacts. Even more importantly, neither of these databases includes symmetry-related chains so that many specific contacts are necessarily missing as they simply are not included for consideration. Our algorithm aims to include only specific contacts in predicted biological complexes, and we take special care to enumerate all contacts observed in the X-ray crystal structures by applying the necessary symmetry operations. Of course, biological applications relying on these databases are mostly interested in the biologically relevant domain interactions rather than crystal contacts.

### Online database of protein complexes

**Figure 5:**
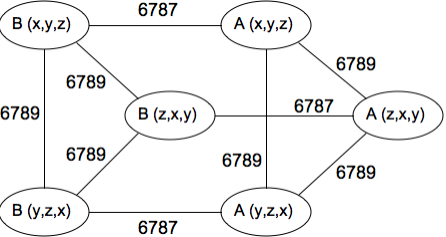
**Schematic graph representation of the predicted homohexamer complex for *E. coli *phosphopantetheine adenylyltransferase (PDB entry 1B6T)**. Nodes represent subunits, denoted by their chain name and symmetry transformation, and edges represent inter-subunit contacts in the complex.

A searchable database of all predicted protein complexes is available online (see Availability and requirements section for URL). Search fields include the PDB entry name, text in the complex or subunit description, interface cluster number, or the Pfam accession number. The complex structures matching the query are presented both explicitly as three-dimensional structures and schematically as graphs in which nodes represent subunits and edges represent contacts (see Figure 5). In addition, all data files are directly available for download by FTP.

## Conclusion

High-resolution structures provide unique information about biological complexes, which is not available by any other experimental or theoretical method. In addition to being the most reliable evidence for the existence of the complex, high-resolution structures give clues about functional mechanisms, elucidate stoichiometry requirements, and allow functional comparisons with orthologs from other species. In many cases a high-resolution structure can be used to guide further experimental work, *e.g*. discovery of small molecules modulating the formation or function of the complex.

The reconstruction of a complete biological subunit proved to be a complex computational problem both due to the amount of the required technical work (*e.g*. many contacts occur outside of elementary crystal cell, so that all symmetries must be carefully analyzed) and due to fundamental difficulties of differentiating between biological and non-biological contacts. Separate questions arise from the presence of incomplete protein complexes. In those cases the crystallized assembly is only part of true biological complex. The PDB BIOMT annotation is known to contain a large number of incorrect annotations, and two other previously developed approaches (PQS [12] and PISA [14]) provide alternative annotations. The validity of this information is very important, as it is utilized in a large number of other databases without modification and without any further critical analysis. These derived databases are used for comparative genomics studies, reconstruction of cellular pathways, *etc*. and the errors introduced at the annotation stage adversely affect all results derived from them.

Our method has demonstrated an almost two-fold reduction of the error rate for predictions on a large set of 435 protein complexes manually assigned through analysis of available biological literature. The error rate for our method was 11% as compared with error rates of 17–18% for PQS and approximately 24% for PISA. There was also a smaller reduction in the error rate for a subset of protein complexes from Ponstingl et al. 2003 from 16% for that method to 13% for the method described here.

Another result of our work is the clustering of similar interfaces. The enforced similarity guarantees that two parts of the interface not only have similar biological sequences, but also have similar spatial arrangements. The groups are created for all contacts found in the PDB (including those which were later classified as non-biological). The availability of interface clusters opens several possibilities for further analysis. For example, it is possible to check the literature for biological evidence of a particular interface by using sources related to all PDB complexes containing it. This could greatly increase our confidence in the annotation of the difficult cases, both on the level of individual interfaces and on the level of complete complexes. Further analysis of these interface groups and how they co-occur in complexes may give insights into the evolutionary history of protein complexes.

An analysis of the contribution of each interface property to the accurate Random Forest prediction of protein-protein interfaces showed that the interface area, number of intramolecular hydrogen bonds, evolutionary conservation, over-represented (and energetically favourable) interface residues and residue-residue contacts, and interface packing density contributed the most. An important advantage of the Random Forest method over other machine learning methods is that it can use all of these diverse properties, without adjusting their relative normalization, in order to make an accurate prediction.

The observed reduction of the error rate is both significant and valuable for applications, but in our opinion it is also surprising that it remains relatively high. In our study we used a comprehensive set of interface characteristics, but obviously more has to be done to understand what governs contacts under biological conditions. Several further directions for study are possible. One of the most interesting would be to combine our machine learning approach with free energy calculations as suggested by the approach used for the PISA database [14]. Another possible direction could involve algorithms to integrate information from several interfaces together with "global" information about complex. In our work we have used BIOMT stoichiometry as such global information, but many problems remains unresolved, in particular the relative weights assigned to global and local components.

Finally, we found relatively little agreement on which Pfam domains form contacts in protein complexes among complexes predicted from our method and those in the iPfam and 3DID databases. We attribute this discrepancy mainly to crystal contacts and missing subunits in the other databases due to ignoring symmetry-related chains. It would be interesting to also compare the set of interacting Pfam domains in the complexes identified by our method with those predicted from yeast two-hybrid data [42].

## Availability and requirements

Protein Interface Server (PInS):  

## Authors' contributions

AB conceived the prediction methodology and carried out the calculations. Both authors participated in the analysis of the results, database website design, and drafting the manuscript. Both authors read and approved the final manuscript.

## Supplementary Material

Additional file 1 **Benchmark set of 435 protein complex structures with prediction results**. The file is a tab-separated table with the following columns: PDB entry; correct oligomeric state; data source (P: Ponstingl et al. 2003, N: Nooren and Thornton 2003, or A: newly added structure); oligomeric state of the complex predicted by our method; oligomeric state of the complex predicted by PQS; total number of subunits in the complex predicted by PISA; and oligomeric state of the PDB BIOMT complex. Click here for file
